# Supplementary material for: The Systems Biology Research Tool: evolvable open-source software
Source: BMC Syst Biol. 2008 Jun 29;2:55. doi: 10.1186/1752-0509-2-55 (PMC2446383; doi:10.1186/1752-0509-2-55)
Supplement: Additional file 1 — SBRT Archive. An archive of the current version of the Systems Biology Research Tool. [file 1752-0509-2-55-S1.zip › sbrt-1.4.0/doc/developers_guide/api/sbrt/shell/text/math/IndexPairFormatV1.html]

IndexPairFormatV1


|  |  |  |
| --- | --- | --- |
| **Overview** \| **Package** \| **Class** \| **Use** \| **Tree** \| **Deprecated** \| **Index** \| **Help** | |  |
| **PREV CLASS**   **NEXT CLASS** | **FRAMES**    **NO FRAMES**     **All Classes** |  |
| SUMMARY: NESTED \| FIELD \| CONSTR \| METHOD | DETAIL: FIELD \| CONSTR \| METHOD |  |


---


## sbrt.shell.text.math Class IndexPairFormatV1<R,C>

```
java.lang.Object
  sbrt.shell.text.math.IndexPairFormatV1<R,C>
```

**Type Parameters:**: `R` - the row index type.: `C` - the column index type.

**All Implemented Interfaces:**: Format, Formatter<IndexPair<R,C>>, IndexPairFormat<R,C>, Parser<IndexPair<R,C>>, SimpleFormat<IndexPair<R,C>>

---

``` public final class IndexPairFormatV1<R,C> extends java.lang.Object implements IndexPairFormat<R,C> ```

This class is a concrete implementation of `IndexPairFormat`.
The string representation of indices used by this class is:
`[RowIndex | ColumnIndex]`.

**Author:**
:   This class was written and documented by
    Jeremiah Wright while in the Wagner lab.

---

**Constructor Summary**

`IndexPairFormatV1(SimpleFormat<R> rowIndexFormat,
SimpleFormat<C> columnIndexFormat)`
  
          Constructs a new index pair format.


| **Method Summary** | |
| --- | --- |
| `<S extends IndexPair<R,C>> java.lang.String` | `format(S indexPair)`             Returns a formatted string representation of the provided index pair. |
| `java.lang.String` | `formatPair(IndexPair<? extends R,? extends C> indexPair)`             Returns a formatted string representation of the provided index pair. |
| `SimpleFormat<C>` | `getColumnIndexFormat()`             Returns the format used for column indices. |
| `SimpleFormat<R>` | `getRowIndexFormat()`             Returns the format used for row indices. |
| `IndexPair<R,C>` | `parse(java.lang.String indexPair)`             Parses the provided string and returns its corresponding index pair. |

**Methods inherited from class java.lang.Object**

`clone, equals, finalize, getClass, hashCode, notify, notifyAll, toString, wait, wait, wait`

**Constructor Detail**

### IndexPairFormatV1

```
public IndexPairFormatV1(SimpleFormat<R> rowIndexFormat,
                         SimpleFormat<C> columnIndexFormat)
```

:   Constructs a new index pair format.

    **Parameters:**: `rowIndexFormat` - the row index formatter.: `columnIndexFormat` - the column index formatter.


**Method Detail**

### getRowIndexFormat

```
public SimpleFormat<R> getRowIndexFormat()
```

:   Returns the format used for row indices.

    :   **Returns:**: the format used for row indices.

---


### getColumnIndexFormat

```
public SimpleFormat<C> getColumnIndexFormat()
```

:   Returns the format used for column indices.

    :   **Returns:**: the format used for column indices.

---


### formatPair

```
public java.lang.String formatPair(IndexPair<? extends R,? extends C> indexPair)
```

:   Returns a formatted string representation of the
    provided index pair.

    :   **Parameters:**: `indexPair` - the index pair to be formatted. **Returns:**: a formatted string representation of the provided index pair.

---


### format

```
public <S extends IndexPair<R,C>> java.lang.String format(S indexPair)
```

:   Returns a formatted string representation of the
    provided index pair.

    :   **Specified by:**: `format` in interface `Formatter<IndexPair<R,C>>`
    :   **Type Parameters:**: `S` - the type of index pair. **Parameters:**: `indexPair` - the index pair to be formatted. **Returns:**: a formatted string representation of the provided index pair.

---


### parse

```
public IndexPair<R,C> parse(java.lang.String indexPair)
```

:   Parses the provided string and returns its
    corresponding index pair.

    :   **Specified by:**: `parse` in interface `Parser<IndexPair<R,C>>`
    :   **Parameters:**: `indexPair` - the string to be parsed. **Returns:**: the index pair corresponding to the provided string.


---


|  |  |  |
| --- | --- | --- |
| **Overview** \| **Package** \| **Class** \| **Use** \| **Tree** \| **Deprecated** \| **Index** \| **Help** | |  |
| **PREV CLASS**   **NEXT CLASS** | **FRAMES**    **NO FRAMES**     **All Classes** |  |
| SUMMARY: NESTED \| FIELD \| CONSTR \| METHOD | DETAIL: FIELD \| CONSTR \| METHOD |  |


---
